# Depression Is Associated With Constipation in Patients With Parkinson's Disease

**DOI:** 10.3389/fneur.2020.567574

**Published:** 2020-12-16

**Authors:** Qin Xiao-ling, Chen Gang, Lu Bo, Li Zai-li, Liu Xue-kui, Li Xue, Shi Ming-yu, Du Yin-zhen, Chen Xu, Gao Dian-shuai

**Affiliations:** ^1^Department of Neurology, Shanghai Eighth People's Hospital, Shanghai, China; ^2^Xuzhou Key Laboratory of Neurobiology, Department of Neurobiology and Anatomy, Xuzhou Medical University, Xuzhou, China; ^3^Department of Gastroenterology, Shanghai Yangsi Hospital, Shanghai, China; ^4^Xuzhou Clinical School of Xuzhou Medical University, Xuzhou, China

**Keywords:** Parkinson's disease, constipation, prodromal stage, serotonergic system, tremor

## Abstract

**Objective:** Constipation is one of the most frequent non-motor symptoms (NMS) in Parkinson's disease (PD), causing great disturbance to patients. The present study investigated the prevalence and the clinical features of constipation in patients with PD and explored the difference between prodromal and clinical constipation of PD.

**Methods:** A total of 186 patients with PD were recruited into this study. Subjective constipation was defined by ROME III criteria. Demographic and PD-related clinical information of the participants were collected. The PD patients were objectively assessed by a spectrum of rating scales of motor symptoms, non-motor symptoms, and quality of life.

**Results:** In total, 51.61% (96/186) of PD patients suffer from constipation. Compared with patients without constipation, the patients with constipation were prone to have restless leg syndrome, depression, and anxiety and have higher scores of the non-motor symptoms scale. Among patients with constipation, 21.88% (21/96) patients had constipation in prodromal stage. Compared with patients with constipation in clinical stage, patients with prodromal constipation had a lower age of constipation onset (56.48 ± 9.63 and 65.26 ± 8.42, χ^2^ = 4.091, *P* < 0.001), longer timespan from constipation onset to motor symptom onset (6.62 ± 3.91 and 3.18 ± 2.13, χ^2^ = −3.877, *P* = 0.001). Patients with prodromal constipation were predominantly tremor onset (χ^2^ = 4.405, *P* = 0.044) and usually had a better quality of life [28 (14.50–37.5) and 40 (25.0–55.0), χ^2^ = 2.011, *P* = 0.046]. Depression was the only risk factor of constipation in PD patients. Body mass index, depression, and anxiety were factors that affected the life quality in patients with constipation.

**Conclusions:** Our results supported the high incidence of constipation in patients with PD and that, in some patients, constipation occurred before the onset of motor symptoms. The specific clinical characteristics of patients with constipation and with prodromal constipation help to make early diagnosis, to discover the relationship between constipation and PD, and to further explore the pathogenesis of this degenerative disease.

## Introduction

Parkinson's disease (PD) is a common neurodegenerative disorder characterized by motor (rigidity, bradykinesia, resting tremor) and non-motor symptoms (autonomic dysfunction, neuropsychiatric disturbance, abnormal sensation, and sleep disorders) (1). Constipation is one of the most frequent non-motor symptoms of gastrointestinal dysfunction in the autonomic system, the prevalence of which has been reported with a wide spectrum ranging from 7 to 71% among different studies, mainly due to the different diagnostic criteria (2). It has been reported that constipation can precede motor symptoms by as much as 20 years, and people with constipation may have a relatively high risk of developing PD (3). In addition, constipation is one of the predicted symptoms listed in the MDS research criteria for prodromal Parkinson's disease, with a relatively high positive likelihood ratio of 2.2 (4). Therefore, the study of constipation in PD patients contributes to understanding the pathogenesis of the disease.

Although progressive dopaminergic denervation is the cardinal pathology in substantia nigra pars compacta (SNpc) of patients with PD, other systems, such as the serotonergic system, are affected as well. Over the last decade, studies have suggested that a progressive and non-linear loss of serotonergic terminals takes place in PD and that serotonergic dysfunction in PD is associated with the development of motor and non-motor symptoms (5–7).

Slow transit in the colon is responsible for chronic constipation in PD (8). Serotonin (5-hydroxytryptamine, 5-HT) mediates the peristaltic movements of the gastrointestinal tract by binding to receptors (especially 5-HT4), stimulating the release of neurotransmitters such as acetylcholine and causing smooth muscle contraction behind the luminal contents and propelling them forward (9). However, serotonergic neurons in the gut wall are critical in regulating constitutive gastrointestinal motility (10). Emerging evidence suggests that the neurodegenerative process in PD starts in the enteric nervous system and spreads *via* the vagus to the lower brainstem and the dopaminergic nigrostriatal system (11). This hypothesis could explain the pathogenesis of constipation and why this symptom precedes the development of PD (12).

Considering the scope of the problem and its impact on the pathophysiology and prognosis of PD, constipation is still a problem that needs to be clarified, and better characterized. In this study, we aimed to investigate the prevalence of constipation in a Chinese population of PD patients and compare the demographic characteristics, property of constipation, and motor symptom and non-motor symptoms between premotor constipation and post-motor constipation in patients with PD.

## Methods

### Patients

A total of 186 idiopathic PD patients were recruited from the clinic or from in-patients at the Department of Neurology, Xuzhou Central Hospital/Clinical Hospital of Xuzhou Medical University from March 2017 to December 2019. The clinical diagnosis of idiopathic PD was determined based on the MDS clinical diagnostic criteria for Parkinson's disease (13). Subjects with other diseases, such as respiratory diseases, urinary system diseases, cardiovascular and cerebrovascular diseases, and primary mental disorders, were excluded (but patients with cognitive disorder were not deliberately excluded). Subjects who were unable to finish the questionnaire were also excluded.

The protocol was approved by the Ethics Committee of Xuzhou Clinical Hospital of Xuzhou Medical University. All participants completed the written informed consents.

### Clinical Assessments

#### Demographic and General Information

A movement disorder specialist clinically evaluated the PD subjects in an “ON” state. Demographic variables, including gender, age, education level, height, weight, and lifestyle (occupational exposure to insecticides/herbicides, smoker, and drinks alcohol), were recorded for all participants. General clinical information, including levodopa equivalent daily dose [LEDD, calculated based on previously reported conversion factors (14)], age of motor symptom onset, disease duration, symptom of onset (tremor or non-tremor), side of onset (left, right, or dual sides), and clinical phenotype were recorded for all participants. If the patients have constipation, the age of constipation onset and years from motor symptom to constipation onset were also recorded. Among these data, age of motor symptom onset and age of constipation onset were defined as self-perceived (subjective) signs. Disease duration was defined as the time from the onset of motor symptoms to the time of the survey.

#### Constipation Assessment

Constipation was defined through the ROME III functional constipation criteria (15). The ROME III functional constipation criteria define constipation as the presence of two or more symptoms (straining for defecation, lumpy/hard stools, sensation of incomplete evacuation, sensation of anorectal obstruction, manual maneuvers, and <3 bowel movements per week) at least 25% of the time for more than 3 months, with onset for at least 6 months. According to the ROME criteria of constipation, the patients in this study were divided into PD with constipation (Cons-PD) and PD with no constipation (nCons-PD) groups. In the Cons-PD group, we further asked whether the constipation occurred before or after the onset of motor symptoms. Patients with constipation before the onset of motor symptoms were referred to as the prodromal stage constipation group (Cons-prodromal-PD), while patients with constipation after the onset of motor symptoms were referred to as the clinical stage constipation group (Cons-clinical-PD). The Patient Assessment of Constipation Quality of Life scale (PAC-QoL) was used to evaluate the life quality of patients with constipation. PAC-QoL is a 28-item self-reported questionnaire, which is divided into four subscales: physical discomfort (items 1–4), psychosocial discomfort (items 5–12), worries and concerns (items 12–23), and satisfaction (items 24–28) (16). Neurologists confirmed the final results.

#### Assessment of Motor and Non-motor Symptoms

The severity of PD was assessed by Hoehn–Yahr (H&Y) stage and the Unified Parkinson's disease rating scale (UPDRS). The motor symptoms of PD patients were evaluated by UPDRS III (17). Onset of motor symptom was divided into tremor and non-tremor. The onset locations were marked as left (left upper or lower limbs), right (right upper or lower limb), and others (bilateral or head). Motor phenotypes were identified based on the ratio of the mean tremor score (sum of items 20 and 21 in the UPDRS III divided by four) to the mean bradykinesia/rigid score (sum of items 22–27 and 31 in the UPDRS III divided by 15). Patients with a ratio >1.0, <0.80, and between 0.80 and 1.0 were classified into the tremor-dominant subtype, akinetic-rigid-dominant subtype, and mixed subtype, respectively (18).

Each participant also completed the Pittsburgh Sleep Quality Index (PSQI), Epworth Sleepiness Scale (ESS), Rapid Eye Movement Sleep Behavior Disorder Screening Questionnaire (RBD-SQ), restless leg syndrome (RLS) diagnosis, Hamilton's Depression Scale (HAMD), Hamilton's Anxiety Scale (HAMA), Mini-mental State Examination (MMSE), Montreal Cognitive Assessment (MoCA), and Parkinson's Disease Non-motor Symptoms Scale (PD-NMSS). The PSQI is a self-report questionnaire that assesses nighttime sleep over a 1-month time period, with a global score ranging from 0 to 21. Higher scores represent poorer subjective sleep quality (19). The ESS is a widely used questionnaire for assessing the general level of daytime sleepiness. The ESS is composed of eight items that address typical day-to-day situations. Each item ranges from 0 to 3 points (0 = would never doze, 3 = high chance of dozing) to yield a total ESS score of 0–24 (lowest to highest sleep propensity). Subjects with ESS >10 were considered as excessive daytime sleepers (EDS), and normal sleep propensity was 0–10 (20). The RBD-SQ is a valuable tool for screening rapid eye movement (REM) sleep behavior disorder (RBD) (21). An RBD score of 5 or greater was defined as probable RBD. A diagnosis of RLS was made according to the RLS diagnostic criteria proposed by the International Restless Legs Syndrome Study Group (IRLSSG) in 2014, which is based on four essential features of the questionnaire after the exclusion of RLS mimics, such as positional discomfort, muscle cramp, venous stasis, vascular claudication, and peripheral neuropathy (22). HAMD and HAMA are frequently used to quantify the severity of depression and anxiety, respectively (23). MMSE and MoCA are the most widely used by frontline physicians to clinically assess cognitive functions. PD-NMSS is a widely used self-administered 30-item instrument for screening the presence of a series of non-motor symptoms and calculating the incidence of each non-motor symptom (24).

## Statistical Analysis

The measurement data are expressed as mean ± SD (standard deviation), and the enumeration data are shown as numbers (rate). Two independent-sample *t*-tests were used to analyze the measurement data of two groups with a normal distribution. Non-normal distribution data were analyzed using non-parametric tests (Mann–Whitney test). The enumeration data were analyzed using χ^2^ test. We used logistic regression analysis to assess the correlations between the different factors and constipation in PD patients. Linear regression analysis was performed to figure out the risk factors of PAC-QoL. *P* < 0.05 was considered as statistically significant.

## Results

### Demographic Data of PD Patients

A total of 186 idiopathic PD patients were ultimately included in this study. There were 95 male patients (55.60%) and 91 female patients (44.40%). The mean age was 67.53 ± 8.52 years old (range: 42–87 years), with a mean disease duration of 68.11 ± 69.59 months (range: 3–420 months).

### Assessment of Constipation in PD Patients

In 186 PD patients, 96 cases (51.61%) were with constipation (Cons-PD), and 90 cases (48.39%) were with no constipation (nCons-PD). Among the 96 PD patients with constipation, 21 cases (21.88%) experienced constipation at prodromal stage or before the onset of motor symptoms (Cons-prodromal-PD), and 75 cases (78.12%) experienced constipation at clinical stage or after the onset of motor symptoms (Cons-clinical-PD).

### Clinical Features of Cons-PD and nCons-PD Groups

The demographic variables and the clinical features of Cons-PD and nCons-PD groups are compared in Table 1. There was no significant difference in age, gender ratio, education level, BMI, and lifestyle between the two groups. In terms of clinical features, there was no difference in LEDD, age of motor symptom onset, disease duration (equivalent to the duration of motor signs), onset location, clinical phenotype, UPDRS III score, and H&Y stage between the two groups. Both the two groups presented more tremor onset symptom; however, the number of patients with tremor onset in the Cons-PD group (63.54%) was significantly higher than that in the nCons-PD group (54.44%) (*P* = 0.043). The prevalence of RLS in the Cons-PD group was significantly higher than in the nCons-PD group (*P* = 0.043), but there were no significant difference in other sleep disorder evaluations, including PQSI score, ESS scores, and RBD. The scores of HAMA and HAMD were significantly higher in the Cons-PD group than in the nCons-PD group (*P* = 0.004, *P* = 0.003, respectively). The score of NMSS in the Cons-PD group was also significantly higher than in the nCons-PD group.

**Table 1:** Demographic and clinical data of Parkinson's disease (PD) patients with or without constipation.

	**PD (*****n*** **=** **186)**		***t*/*Z*/χ^**2**^**	***P***
	**Cons-PD (*n* = 96, 51.61%)**	**nCons-PD (*n* = 90, 48.39%)**		
Age (years)	67.93 ± 8.08	67.13 ± 9.03	−0.63	0.53
Sex, female (%)	44 (45.8%)	47 (52.2%)	0.759	0.384
Education (years)	7.14 ± 4.55	7.06 ± 4.43	−0.121	0.904
Height	1.64 ± 0.77	1.63 ± 0.71	−1.416	0.159
Weight	64.01 ± 10.68	62.55 ± 9.99	−0.961	0.338
BMI	23.63 ± 3.37	23.55 ± 3.07	−0.176	0.861
**Lifestyle**				
Occupational exposure to insecticides/herbicides	6 (6.3%)	1 (1.1%)	3.387	0.066
Smoke	14 (14.6%)	11 (12.2%)	0.223	0.637
Drink Chinese liquor	12 (12.5%)	7 (7.8%)	1.129	0.288
LED (mg)	412.68 ± 224.64	414.80 ± 238.70	0.062	0.950
Disease duration (months)	48 (24–84)	48 (17–90)	−0.608	0.543
Age of motor symptom onset	62.30 ± 9.08	61.70 ± 10.08	−0.428	0.669
**Onset symptom (case, %)**				
Tremor	41 (42.7%)	51 (56.7%)	3.621	0.057
Non-tremor	55 (57.3%)	39 (43.3%)		
***Onset location (case, %)***				
Left	52 (54.17%)	41 (45.6%)		
Right	36 (37.5%)	43 (47.8%)	2.016	0.365
Dual side	8 (8.3%)	6 (6.7%)		
***Clinical phenotype (case, %)***				
TD	22 (22.9%)	30 (33.3%)	3.719	0.156
AR	29 (30.2%)	18 (20.0%)		
Mixed	45 (46.9%)	42 (46.7%)		
H&Y (on-stage)	2 (2–3)	2 (1–3)	−1.182	0.237
UPDRS-I	10.43 ± 7.88	8.79 ± 7.12	−1.484	0.140
UPDRS-II	18.47 ± 13.05	16.76 ± 13.92	−0.556	0.580
UPDRS-III	33.39 ± 24.81	28.41 ± 24.82	−1.366	0.174
UPDRS-IV	0 (0–3.75)	0 (0–0)	−1.241	0.214
PQSI (scores)	8 (5–10)	7 (3–11.50)	−1.677	0.094
ESS (scores)	7.26 ± 6.19	6.22 ± 5.93	−1.166	0.245
RBD, case (%)	26 (27.1%)	29 (32.2%)	0.589	0.443
RLS, case (%)	28 (29.2%)	15 (16.7%)	4.084	0.043
HAMA (scores)	7 (2.25–14.87)	2.50 (0.00–8.00)	−2.864	0.004
HAMD (scores)	10 (4.25–21.75)	3 (0.25–10.00)	−3.021	0.003
MoCA	14.56 ± 6.95	16.32 ± 8.41	0.993	0.324
MMSE	23.56 ± 4.64	23.39 ± 7.21	−0.118	0.907
NMSS	46.50 (18.50–80.0)	19 (7–70.0)	−1.514	0.130

*PD, Parkinson's disease; Cons-PD, PD with constipation; nCons-PD, PD with no constipation; BMI, body mass index; LED, daily levodopa-equivalent dose; TD, tremor-dominant subtype; AR, akinetic-rigid subtype; Mixed, mixed subtype; H&Y, Hoehn–Yahr stage; UPDRS-I, United Parkinson's Disease Ranking Scale Part I; UPDRS III, United Parkinson's Disease Ranking Scale Part III; PSQI, Pittsburgh Sleep Quality Index; ESS, Epworth Sleepiness Scale; RBD, rapid eye movement (REM) sleep behavior disorder; RLS, restless legs syndrome; HAMA, Hamilton's Anxiety Scale; HAMD, Hamilton's Depression Scale; MMSE, Mini-Mental State Examination; MoCA, Montreal Cognitive Assessment*.

### Clinical Features of Cons-Prodromal-PD and Cons-Clinical-PD Groups

The demographic variables and the clinical features of Cons-prodromal-PD and Cons-clinical-PD groups are compared in Table 2. There was no significant difference in age, gender ratio, education level, body mass index (BMI), and lifestyle between the two groups. In terms of clinical features, there were no differences in LEDD, age of motor symptom onset, disease duration, onset location, clinical phenotype, UPDRS III score, and H&Y stage between the two groups. However, the age of constipation onset was significantly lower in the Cons-prodromal-PD group than in the Cons-clinical-PD group (56.48 ± 9.63 and 65.26 ± 8.42, respectively; χ^2^ = 4.091, *P* < 0.001). The timespan from constipation onset to motor symptom onset in Cons-prodromal-PD was longer than in Cons-clinical-PD (6.62 ± 3.91 and 3.18 ± 2.13, respectively, χ^2^ = −3.877, *P* = 0.001). Cons-prodromal-PD was predominantly tremor onset, while Cons-clinical-PD was non-tremor onset (χ^2^ = 4 045, *P* = 0.044). There were no differences between the two groups in ESS, RBD, PQSI, RLS, MMSE, and MoCA. The HAMA and HAMD scores, which manifested a significant difference between the Cons-PD and the nCons-PD groups, were not different between Cons-prodromal-PD and Cons-clinical-PD. There was no difference in NMSS between the two groups. PAC-QoL in the Cons-prodromal-PD group was lower than in the Cons-clinical-PD group [28 (14.50–37.5) and 40 (25.0–55.0), χ^2^ = 2.011, *P* = 0.046].

**Table 2:** Demographic and clinical data of patients with constipation in prodromal and clinical stages of Parkinson's disease (PD).

	**PD (*****n*** **=** **96)**		***t*/*Z*/χ^**2**^**	***P***
	**Cons-prodromal-PD (*n* = 21, 21.88%)**	**Cons-clinical-PD (*n* = 75, 78.12%)**		
Age (years)	68.38 ± 8.59	67.80 ± 7.98	0.29	0.773
Sex, female (%)	12 (57.1%)	32 (42.7%)	1.385	0.239
Education (years)	6.57 ± 5.34	7.29 ± 4.33	−0.640	0.524
Height	1.64 ± 0.07	1.65 ± 0.07	−0.392	0.696
Weight	63.43 ± 10.24	64.17 ± 10.86	−0.281	0.779
BMI	23.54 ± 2.98	23.66 ± 3.49	−0.146	0.884
***Lifestyle***				
Occupational exposure to insecticidal/ herbicides	0 (0)	6 (8.0%)	1.192	0.181
Smoke	3 (14.29%)	11 (14.67%)	0.002	0.965
Drink Chinese liquor	2 (9.52%)	10 (13.33%)	0.218	0.641
LED (mg)	433.10 ± 197.05	406.96 ± 232.68	0.469	0.640
Disease duration (months)	84 (36–102)	48 (24–72)	−0.884	0.377
Age of motor symptom onset	63.10 ± 9.79	62.08 ± 8.90	0.451	0.635
Age of constipation onset	56.48 ± 9.63	65.26 ± 8.42	4.091	0.001
Timespan from constipation onset to motor symptom onset (years)	6.62 ± 3.91	3.18 ± 2.13	−3.877	0.001
***Initial symptom (case, %)***				
Tremor	13 (61.90%)	28 (37.33%)	4.045	0.044
Non-tremor	8 (38.10%)	47 (62.67%)		
***Onset location (case, %)***				
Left	9 (42.9%)	43 (57.3%)	1.983	0.371
Right	9 (42.9%)	27 (36.0%)		
Dual side	3 (14.3%)	5 (6.7%)		
***Clinical phenotype***				
TD	6 (28.6%)	16 (21.3%)	1.657	0.437
AR	4 (19.0%)	25 (33.3%)		
Mixed	11 (52.4%)	34 (45.3%)		
H&Y (on-stage)	3 (2–5)	2 (2–3)	−1.088	0.276
UPDRS-I	10.81 ± 8.30	10.32 ± 7.81	0.25	0.803
UPDRS-II	19.78 ± 10.84	18.04 ± 13.87	0.342	0.734
UPDRS-III	32.10 ± 19.73	33.75 ± 16.16	0.268	0.789
UPDRS-IV	0 (0–5)	0 (0–3)	−0.022	0.983
PSQI (scores)	9 (8–12)	7 (4–10)	−0.786	0.432
ESS (scores)	8.29 ± 5.89	6.97 ± 6.28	−0.268	0.789
RBD, case (%)	7 (33.3%)	19 (25.3%)	0.532	0.466
RLS, case (%)	5 (23.8%)	23 (30.7%)	0.373	0.541
HAMA (scores)	10 (2.5–21.5)	6 (2–12)	−1.762	0.078
HAMD (scores)	17 (4.5–25.5)	10 (4–18)	−1.492	0.136
MMSE	22.89 ± 4.54	23.78 ± 4.74	−0.493	0.626
MoCA	11.11 ± 7.46	15.70 ± 6.51	1.769	0.086
NMSS	56(22–84.0)	37(18–82)	−0.311	0.756
PAC-QoL	28(14.50–37.5)	40(25.0–55.0)	2.011	0.046

*PD, Parkinson's disease; Cons-prodromal-PD, PD with constipation at prodromal stage; Cons-clinical-PD, PD with constipation at clinical stage; BMI, body mass index; LED, daily levodopa-equivalent dose; TD, tremor-dominant subtype; AR, akinetic-rigid subtype; Mixed, mixed subtype; H&Y, Hoehn–Yahr stage; UPDRS-I, United Parkinson's Disease Ranking Scale Part I; UPDRS III, United Parkinson's Disease Ranking Scale Part III; PSQI, Pittsburgh Sleep Quality Index; ESS, Epworth Sleepiness Scale; RBD, rapid eye movement (REM) sleep behavior disorder; RLS, restless legs syndrome; HAMA, Hamilton's Anxiety Scale; HAMD, Hamilton's Depression Scale; MMSE, Mini-Mental State Examination; MoCA, Montreal Cognitive Assessment*.

### Risk Factors of Cons-PD

Logistic regression analysis was performed to figure out the risk factors of PD-C in Table 3. Eventually, it was found that only HAMD score was the risk factor of constipation in PD patients (odds ratio, OR = 1.037; *P* < 0.01).

**Table 3:** Regression analysis of factors associated with constipation in patients with Parkinson's disease.

**Variables**	**Beta**	**SE**	**Wald**	***P***	**OR**	**95% CI**
Hamilton's depression scale	0.036	0.017	4.352	0.037	1.037	1.002–1.072

### Risk Factors of PAC-QoL

Linear regression analysis was performed to figure out the risk factors of PAC-QoL in Table 4. Eventually, it was found that BMI, HAMD, and HAMA scores were the risk factors of PAC-QoL in PD patients with constipation (OR = 1.091; *P* < 0.01).

**Table 4:** The association between some variables and patient assessment of constipation quality of life scale.

**Variables**	**Beta**	**95% CI**	***t***	***P***
Body mass index	2.096	0.428–3.765	2.503	0.015
Hamilton's depression scale	0.593	0.086–1.099	2.332	0.022
Hamilton's anxiety scale	0.905	0.200–1.610	2.558	0.013

## Discussion

The clinical symptoms of PD are divided into motor and non-motor symptoms. Among non-motor symptoms, constipation is unanimously regarded as one of the most relevant and difficult to treat. In this study, 51.61% PD patients had constipation, in accordance with previous studies reporting 50 to 80% of constipation in PD patients (2) but much higher than the estimated global prevalence of chronic constipation of 14% (25). Thus, the study confirmed the high prevalence of constipation among patients with PD.

We did not find differences between Cons-PD and nCons-PD in demographic data and lifestyle, including sex, education, exposure to insecticides/herbicides, drinking Chinese liquor, and smoking. Although it has been reported that women are more prone to experience chronic constipation in general subjects (26) and that men are more likely to suffer from constipation (27), we did not find these in patients with PD in this study.

We further found that Cons-PD patients tended to have a statistically high percentage of tremor onset phenotype, and they were more prone to be accompanied by sleep disorders (RLS in particular), anxiety, depression, as well as more other non-motor symptoms. There was no significant difference in the severity of motor symptom, disease duration, age of motor symptom onset, onset location, and clinical phenotype between these two groups.

Although we did not complete a statistical analysis of Medopa dosage alone, this study showed no difference between LEDs in Cons-PD and nCons-PD groups, being consistent with Frazzitta et al. (28) but contrary to Zhang and Liu's (29) findings. The possible lack of correlation between LED and constipation is worthy of consideration as some authors have previously attributed constipation to dopaminergic treatment (30). As to cognitive function, marked as MoCA and MMSE, no difference was found between these two groups.

We retrospectively surveyed the timespan between onset of constipation and onset of motor symptoms to investigate the clinical features between PD patients with prodromal stage constipation and clinical stage constipation. Our data showed that there were 21.88% of PD patients having constipation before the onset of motor symptoms, that is, the prodromal stage. The result was similar to but slightly lower than those of previous studies, which reported the prevalence of constipation before the onset of motor symptoms as ranging from 28.19 to 87% (29). Compared with clinical-stage-constipation PD patients, prodromal-stage-constipation PD patients were dominated by tremor onset phenotype and characterized with younger constipation onset age and longer timespan from constipation onset to motor symptom onset but with better quality of life. On the contrary, the clinical-stage-constipation PD patients were dominated by non-tremor onset phenotype and presented with older constipation onset age and shorter timespan from constipation onset to motor symptom onset but with worser quality of life. There were no differences in other variables, including demographic data, lifestyle, motor symptoms (onset location, phenotype, severity, etc.), anti-parkinsonian medicament (LED), sleep disorders (RLS, etc.), depression or anxiety, and cognitive functions, between these two groups. When we finally investigated the influencing factors of the life quality of PD patients with constipation, we found that BMI, HAMD, and HAMA were the main factors affecting the quality of life of PD patients with constipation.

Correlation analysis showed that HAMD was positively associated with constipation, suggesting that HAMD might be a risk factor for constipation in PD patients. Considering the small sample size of patients with prodromal constipation, we did not analyze the risk factors of prodromal-stage-constipation PD patients.

We spotlighted in this study that depression was an independent risk factor of constipation, and PD patients with prodromal stage constipation were predominantly tremor onset. Since the underlying clinical implications and mechanisms are unclear, studying and discovering the underlying mechanisms becomes interesting and challenging.

It is generally believed that the pathological feature of PD is the degeneration of dopaminergic neurons in the substantia nigra striatum, together with intraneuronal Lewy bodies and Lewy neurites containing aggregated alpha-synuclein (31). However, the serotonergic system that originated in the raphe nucleus of the brainstem also degenerated. Autopsy studies found the loss of serotonergic cell bodies containing Lewy aggregates in the raphe nucleus (32), as well as the complete absence of serotonergic markers in the cortical and the subcortical structures that receive raphe projections (33). In addition, Braak et al. also found that Lewy pathology in PD occurs in the dorsal raphe nucleus, before the substantia nigra (34). Consistent with post-mortem findings, the serotonin transporter binding measured *in vivo* in patients with PD was significantly reduced (5, 6).

Raphe serotonergic system could contribute to the occurrence of tremor and some non-motor symptoms. Clinical studies have shown that striatal dopamine is relatively spared in patients with the tremor-dominant PD, that rest tremor is related more to serotonergic raphe nuclei deficiency than striatal dopamine (35), and that the severity of resting tremor is also related more to reduced raphe serotonin transporter than dopamine transporter (6, 36). Therefore, disruption of the serotonergic system could contribute to the occurrence of tremor. On the basis of these studies, the fact that prodromal-stage-constipation PD patients were dominated by tremor onset phenotype in the current study might suggest a possible correlation between prodromal constipation of PD and the disruption of the raphe serotonergic system.

Depression is one of the earliest non-motor symptoms in PD, with an incidence of approximately 30% (37). Clinical studies showed that raphe serotonin transporter was related to depression (7, 38) and that accumulation of phosphorylated alphasynuclein occurring in the raphe nuclei leads to depression (34), indicating that 5-HT depletion is one of the mechanisms underlying depression. It is not depression that causes PD, but a common pathology that leads to damage and dysfunction not only of the dopaminergic system but also of the serotonergic systems (39). Thus, depression may occur as a prodromal symptom or at any stage of the disease, and the prodromal depression symptom may be more related to serotonergic system damage than to dopaminergic system damage.

Sleep disorder is another common non-motor symptom in PD, usually classified into insomnia, excessive daytime sleepiness, rapid eye movement sleep behavior disorder, restless legs syndrome, and sleep-disordered breathing (40). There have been reports of the raphe serotonergic system being associated with sleep disorders (41). Four types of sleep disorders were investigated in this study, but only RLS was found to be associated with constipation in PD. In China, the prevalence of RLS in PD ranged from 8.41 to 34.85%. The pathogenesis of RLS in PD is still inconclusive. There is pathological evidence of nigrostriatal dopaminergic system dysfunction in PD and RLS. However, a FP-CIT SPECT study suggested that there might be a non-linear association between dopaminergic dysfunction and RLS (42). In addition, the positive association between RLS and depression (43) and between RLS and risk of suicide and self-harm (44) raises the possibility of a non-dopaminergic mechanism, especially serotonergic mechanism, in RLS as well.

5-HT was first discovered to be present and synthesized in the central nervous system (CNS), but we now know that 5-HT is vastly secreted in the bowel and acquired by platelets, becoming circulating or enteric, making the content of 5-HT in CNS negligible. Much attention was focused on the role of brain 5-HT in the past, but the function of peripheral or intestinal 5-HT has been increasingly concerned and understood. The American Gastroenterological Association classifies primary constipation into three groups on the basis of colonic transit time and anorectal function: normal-transit constipation, slow-transit constipation, and outlet dysfunction. Studies have shown that the colonic transit time in PD patients is twice that of the control group, and researchers considered that constipation in PD patients is related to the decrease of colonic transit function (8). The peristaltic reflex is a fundamental manifestation of propulsive motility in the gut, consisting of oral contraction and aboral relaxation, which occurs in response to elevations of intraluminal pressure. Enterochromaffin cells have classically been regarded as pressure sensors, which secrete 5-HT to initiate peristaltic reflexes (45) and mediate the peristaltic movements of the gastrointestinal tract by binding to receptors (especially 5-HT4), stimulating the release of neurotransmitters such as acetylcholine, causing smooth muscle contraction behind the luminal contents, and propelling them forward (9). The 5-HT content of the enteric nervous system is vanishingly small in comparison to that of enterochromaffin cells. However, deletion of tryptophan hydroxylase 2, which in the gut is restricted to serotonergic neurons, slows down the total gastrointestinal transit time, small intestinal propulsion, and colonic motility, demonstrating the idea that neuronal 5-HT is more important for constitutive gastrointestinal motility than that of enterochromaffin cells (46).

Our study has some limitations. First, it is a retrospective clinical study in which the information of chronology of motor and non-motor symptoms were collected through patients' recall. Although most clinical studies have adopted this method of information collection, recall bias might partially affect the accuracy of our results. Secondly, the lack of a healthy control group may exist when asking about non-motor symptoms. Thirdly, RLS is usually diagnosed by sleep or neurology experts with face-to-face interview. Using the questionnaire for RLS diagnosis may have included RLS mimics and potentially resulted in a high prevalence. Fourthly, no bowel transit time or stool examinations were performed when evaluating constipation, which might lead to a lack of objectivity and quantification of the results. Finally, since it is a single-center retrospective study, the results still requires to be testified by more studies, including multicenter clinical studies.

With limited literature search capabilities, we have not found out reports on the relationship between the raphe serotonergic system and constipation symptom in patients with PD and between the raphe serotonergic system and enteric serotonergic neurons in constipation patients with PD. In the current study, we demonstrated that depression was an independent risk factor of constipation, and PD patients with prodromal stage constipation were predominantly tremor onset. The differences in the results of various studies are related to various factors such as race, population, sample size, diagnostic definition, etc. This requires researchers to further expand the sample size and unify various concepts and evaluation standards in a future study. On the basis of the present study, we are interested in and will investigate the possible relationship between disruption of serotonergic neurons, either in raphe or in gut, and constipation in patients with PD in future studies.

## Data Availability Statement

All datasets generated for this study are included in the article/supplementary material.

## Ethics Statement

The studies involving human participants were reviewed and approved by the Ethics Committee of Xuzhou Clinical Hospital of Xuzhou Medical University approved this study. The patients/participants provided their written informed consent to participate in this study. Written informed consent was obtained from the individual(s) for the publication of any potentially identifiable images or data included in this article.

## Author Contributions

QX and GD designed the study. CG, LB, LZ, LiX, SM, and DY collected the data. LiuX did the statistical analysis. QX and GD analyzed the data and drafted the manuscript. CX participated to draft and revise the manuscript. All authors wrote and revised the article.

## Conflict of Interest

The authors declare that the research was conducted in the absence of any commercial or financial relationships that could be construed as a potential conflict of interest.
